# Linking Autism Risk Genes to Disruption of Cortical Development

**DOI:** 10.3390/cells9112500

**Published:** 2020-11-18

**Authors:** Marta Garcia-Forn, Andrea Boitnott, Zeynep Akpinar, Silvia De Rubeis

**Affiliations:** 1 Seaver Autism Center for Research and Treatment, Icahn School of Medicine at Mount Sinai, New York, NY 10029, USA; marta.garcia-forn@mssm.edu (M.G.-F.); andrea.boitnott@utsouthwestern.edu (A.B.); za637@nyu.edu (Z.A.); 2 Department of Psychiatry, Icahn School of Medicine at Mount Sinai, New York, NY 10029, USA; 3 The Mindich Child Health and Development Institute, Icahn School of Medicine at Mount Sinai, New York, NY 10029, USA; 4 Friedman Brain Institute, Icahn School of Medicine at Mount Sinai, New York, NY 10029, USA; 5 Department of Psychology, College of Arts and Sciences, New York University, New York, NY 10003, USA

**Keywords:** autism spectrum disorder, cortical development, mouse models

## Abstract

Autism spectrum disorder (ASD) is a prevalent neurodevelopmental disorder characterized by impairments in social communication and social interaction, and the presence of repetitive behaviors and/or restricted interests. In the past few years, large-scale whole-exome sequencing and genome-wide association studies have made enormous progress in our understanding of the genetic risk architecture of ASD. While showing a complex and heterogeneous landscape, these studies have led to the identification of genetic loci associated with ASD risk. The intersection of genetic and transcriptomic analyses have also begun to shed light on functional convergences between risk genes, with the mid-fetal development of the cerebral cortex emerging as a critical nexus for ASD. In this review, we provide a concise summary of the latest genetic discoveries on ASD. We then discuss the studies in postmortem tissues, stem cell models, and rodent models that implicate recently identified ASD risk genes in cortical development.

## 1. Introduction

Autism spectrum disorder (ASD) is a neurodevelopmental disorder estimated to affect up to ~1% of the population. The condition is clinically diagnosed by persistent deficits in social communication and interaction, and the presence of repetitive behaviors and/or restricted interests. ASD severity varies greatly and can evolve along different trajectories over time [1,2,3]. Along with ASD severity, cognitive development and language contribute to an individual’s adaptive behavior, as ~30% of affected individuals are minimally verbal [4]. Further, ASD can co-occur with other neurodevelopmental manifestations [e.g., intellectual disability (ID), attention deficit hyperactivity disorder (ADHD)], neurological disorders (e.g., hypotonia, epilepsy, movement disorders), and medical co-morbidities (e.g., congenital anomalies, gastrointestinal problems) [5]. Over the past few years, large-scale genomic studies have brought to light hundreds of genetic loci conferring risk. Through orthogonal transcriptomic datasets and follow-up analyses in cell and animal models, studies are uncovering the functional convergence of risk genes on biological processes. One such process is the development of the neocortex.

In this review, we link recent genetic findings in large-scale ASD genomic studies to functional and mechanistic observations in stem cell models and rodent models. We begin by reviewing the current knowledge of the risk architecture, with a specific focus on the contribution of sequencing studies. We then discuss the most recent findings in human neuroanatomical studies and transcriptomic analyses from human postmortem samples, supporting the importance of cortical dysregulation in ASD pathophysiology. We then focus on the most recent discoveries of the mechanisms and pathways surfacing from manipulations of highly penetrant ASD risk genes in rodent models. The specific genes discussed in each section do not create an exhaustive list of all the ASD risk genes found to be involved in cortical development. We specifically selected autosomal dominant risk genes emerging from the latest exome sequencing studies on ASD [6,7,8] and prioritized the discussion of findings published within the past 3 years. We also included a discussion of known and emerging X-linked ASD risk genes with new evidence implicating them in cortical development.

## 2. ASD Risk Architecture

Enormous progress has been made in our understanding of ASD risk architecture. It is now clear that at least half of the difference in susceptibility amongst individuals depends on genetic variants that are common in the population (common variation, in the form of single-nucleotide polymorphisms or SNPs) [9]. Each of these SNPs has a small effect size, but their additive action determines the difference in liability within the population [9]. While population-based studies [9] and twin studies [10,11,12,13] have provided compelling evidence for a role of common variation in ASD risk, the reproducibility of candidate loci has been hampered by the small sample size (and consequent limited statistical power) of early genome-wide associated studies (GWAS) [14,15,16]. Recently, a much larger GWAS meta-analysis on >18,000 ASD cases contrasted to >27,900 ancestry-matched controls has pinpointed 5 genome-wide significant loci [17].

In parallel, early studies using microarrays [14,18,19,20,21,22,23,24], whole-exome sequencing (WES) on increasingly larger cohorts [6,7,8,25,26,27,28,29,30,31,32,33,34,35,36,37], and more recently whole-genome sequencing (WGS) [38,39,40] have shown the significant contribution of rare genetic variation. Collectively, these efforts have led to the identification of hundreds of genes that when impacted by rare and disruptive genetic variants confer risk to ASD. These variants can be deletions or duplications of intragenic regions, entire genes, or multiple genes (copy number variation, CNV) [14,18,19,20,21,22,23,24,33,34,35]. Alternatively, they can impact as little as one nucleotide (single-nucleotide variation, SNV) or a few nucleotides (indels) [6,7,8,25,26,27,28,29,30,31,32,41]. CNV and SNV/indels conferring risk can be autosomal dominant [6,7,8,25,26,27,28,41], autosomal recessive [25,29], X-linked [25], or mosaic [30,31,32,42]. High-penetrance, autosomal dominant genes, in particular, have been successfully identified by sequencing ASD families, specifically trios (one affected individual and their unaffected parents) and/or quads (one affected individual, their unaffected parents, and their unaffected sibling) [6,7,8,26,28,37,41,43]. To associate discrete genes to ASD risk, early analyses [26,27,28,37] have harnessed the rarity of disruptive variants arising de novo, namely incurring in the parental gametes—in most case paternal [43,44]—and therefore detected only in the affected individual and not in their parents. These studies sequenced ~1000 ASD families and yielded 9 risk genes [26,27,28,37]. Beyond the obvious gain in power due to a larger sample size, newer analyses [6,8,41] have further boosted gene discovery by using refined association frameworks that go beyond de novo variation [45] and/or exploiting evolutionary constraint scores [8,46,47,48]. For example, a recent sequencing study has integrated de novo, inherited, and case-control variation from >35,000 exomes, >11,000 of which from individuals with an ASD diagnosis, and found 102 risk genes [8]. In addition to exome sequencing, genome sequencing is being harnessed to understand the role of rare non-coding variation in liability. A study on genomes from ~1900 quads showed that de novo mutations in promoter regions, especially in evolutionarily conserved binding sites for transcription factors located distally to the transcription start site, contribute to ASD risk [40]. Further, WGS on >2300 families with more than one child affected have been instrumental in identifying rare high-risk inherited variants, which have been elusive due to their modest effect size [38].

Analyses are now probing the interaction between rare and common variation in shaping risk architecture [49,50]. The overlap between loci identified by GWAS [17] and genes pinpointed by exome sequencing [8] is limited to one gene (*KDM5B*), likely due to power limitations [8].

Non-genetic risk factors, and in some cases their interactions with genetic factors, are also emerging [51,52]. One of the most replicated associations for ASD is advanced paternal age, with increased risk in the offspring of fathers in their mid-to-late 30s, further increasing with more advanced paternal ages [52,53,54,55]. The rate of de novo mutations in sperm increases with paternal age [43,44,56,57,58,59], and most de novo mutations found in ASD studies are of paternal origin [26,27]. However, studies based on simulated [60] and empirical [59] data indicate that de novo variation is likely not the major driver for the association between advanced paternal age and ASD risk. Rather, the common polygenic risk for adverse psychiatric outcomes might contribute to later age of childbearing [60,61].

## 3. ASD Risk Converging on Cortical Development—Evidence from Human Studies

Gene discovery has brought major advances in our understanding of the biological processes and pathways associated with risk. ASD risk genes implicated by de novo variation cluster to two main biological processes: synaptic function and gene expression, most prominently chromatin remodeling [6,8]. *KDM5B*, the gene with both GWAS [17] and sequencing signal [8], functions in chromatin remodeling. Some evidence for the role of the cytoskeleton has also begun to surface from de novo [8] and rare inherited [38] variation.

Transcriptomic analyses on postmortem brains sampled from unaffected individuals [62,63,64] or ASD cases contrasted with controls [65,66,67,68,69] have been instrumental to dissect the regions and developmental windows most vulnerable for ASD risk. These studies have shown that ASD genes are often co-expressed during brain development. Further, the spatiotemporal expression patterns of the risk genes implicate the mid-fetal development of the neocortex as a major nexus of risk. The master regulators of this concerted co-expression are also beginning to emerge, including the chromodomain helicase DNA binding protein 8 (*CHD8*) and T-Box brain transcription factor 1 (*TBR1*) [26,27,28,37,70,71,72,73,74,75,76,77]. For example, there is a significant enrichment of risk genes amongst the CHD8-bound genes identified in human neural stem cells (NSCs), mouse developing cortices, and human mid-fetal brain tissues [71,72] and the genes found differentially expressed in *Chd8*-depleted human and mouse progenitors [71,72,73,74,75]. Beyond transcriptional co-regulation, interesting findings are also emerging at the level of post-transcriptional regulation. First, postmortem brains from individuals with ASD show alterations in RNA editing, splicing, and isoform usage [65,66,68,69]. Second, ASD risk genes are enriched in targets of post-transcriptional regulators that are in turn encoded by ASD risk genes, including RNA-binding proteins FMRP (Fragile X Mental Retardation Protein) [6], CELF4 (CUGBP Elav-like family member 4) [8,78], and RBFOX1 (RNA Binding Fox-1 Homolog 1) [6,65,79]. Importantly, many of the mRNAs with altered splicing are targets of the splicing regulator RBFOX1, and 6 genes with isoform-level changes in ASD postmortem brains encode splicing factors [65]. These intriguing observations pose some risk genes as master regulators of downstream risk genes.

Within the cortex, single-cell RNA sequencing (scRNAseq) studies provide more granular resolution on cell types involved. Analyses on scRNAseq data from four fetal samples at mid-fetal development (post-conception weeks 17–18) show that ASD risk genes are predominantly expressed in developing glutamatergic neurons, both upper and lower layers, with interesting exceptions of genes highly expressed in oligodendrocyte precursors and pericytes [64]. Interestingly, ID risk genes are enriched not only in glutamatergic neurons but also in the radial glia (RG) [64]. By performing single-nucleus RNAseq on the prefrontal and anterior cingulate cortices of 15 ASD cases without co-morbid ID and 16 matched controls, Velmeshev and colleagues identified cell-type specific changes in the expression of >500 genes [67]. Interestingly, the degree of differential expression in upper-layer glutamatergic neurons or microglia was the most predictive of clinical severity [67]. Analyses of scRNAseq in human specimens have also the potential to identify species-specific changes. For example, outer radial glia (oRG) is a subtype of radial glia located in the outer subventricular zone (oSVZ) in gyrencephalic brains, such as those of primates and ferrets. The oRGC population is highly abundant in gyrencephalic brains, while it is minimal in lissencephalic species, such as rodents. This indicates that this specific type of progenitors is important for the generation of cortical folds (gyrus and sulci). Interestingly, two ASD risk genes (*GFAP* and *TCF7L2*) [8] are amongst the genes proposed to mark oRG specificity based on scRNAseq on the human cortex at gestational weeks 16–18 [80].

In parallel to sequencing studies, neuroimaging and neuroanatomical studies have provided support for structural changes in cortical development in individuals diagnosed with ASD. For example, a study on postmortem brains from 11 affected children and 11 unaffected controls in the same age range revealed focal patches of altered laminar cytoarchitecture in the prefrontal and temporal cortices of the ASD group [81]. Additional evidence for the role of cortical dynamics in ASD risk come from a prospective neuroimaging study that followed longitudinally 106 high-risk and 42 low-risk infants, whereby risk was defined based on the relationship to an older affected or unaffected sibling. Of the 106 high-risk infants, the 15 that went on to meet diagnostic criteria for ASD at 24 months displayed hyperexpansion of the cortical surface between 6 and 12 months of age, followed by a brain volume overgrowth between 12 and 24 months [82]. Further, a recent study has found that the expression of *TBR1* overlaps with that of visuomotor integrators in the visual and motor cortices, showing the relevance of gene expression co-regulation at a circuit level [83].

## 4. ASD Risk Converging on Cortical Development—Evidence from Cellular and Rodent Models

**Figure 1:**
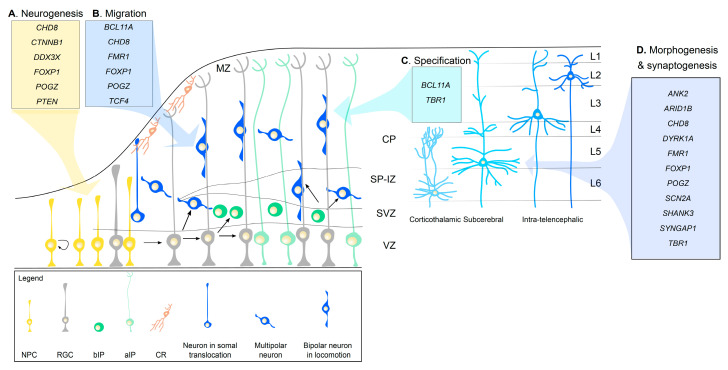
Schematic representation of ASD risk genes involved in corticogenesis reviewed here. Migrating neurons and laminar specific neurons are indicated in different blue tones. NPC, neural progenitor cells; RGC, radial glial cells; bIP, basal intermediate progenitors; aIP, apical intermediate progenitors; CR, Cajal-Retzius cells; MZ, marginal zone; CP, cortical plate; SP-IZ, subplate-intermediate zone; SVZ, subventricular zone; VZ, ventricular zone; L1-6, layers 1-6. Interneurons, cortical afferents, and gliogenesis are not shown for simplicity.

During corticogenesis, a sequence of cellular events occurs to shape the developing cortex [84,85] (Figure 1). The first event to take place is cortical neurogenesis, at embryonic day (E) 10 in mice (33 in human). During this process, neuroepithelial progenitor cells (NPCs), which had previously undergone rapid expansion through symmetrical divisions lining the ventricular zone (VZ), transform into radial glial cells (RGCs). RGCs divide asymmetrically. In each division, RGCs give rise to another RGC, and a postmitotic neuron or an intermediate progenitor (IP). Apical IPs (aIPs) will stay at the VZ, while basal IPs (bIPs) will migrate to the SVZ. IPs divide symmetrically to form two identical neurons. Later in development, RGCs will also give rise to astrocytes and oligodendrocytes. Postmitotic neurons derived from RGCs are excitatory glutamatergic neurons called projection neurons, which project inside the cortex or to other brain regions. These neurons acquire a laminar distribution across the cortex, and morphogenesis and synaptogenesis begin to define their morphology and physiology. Inhibitory GABAergic interneurons do not develop from RGCs, but migrate from the ganglionic eminences, as discussed below.

In the next sections, we will discuss the evidence in rodent models implicating high-risk ASD genes [6,7,8,38] in cortical development. We note that cortical alterations have also emerged in mouse [86,87,88] and stem cell models (reviewed in [89]) of CNVs associated with ASD risk. We will not discuss the circuit changes and behavioral outcomes of the rodent models with mutations in ASD risk genes, which are discussed elsewhere [90,91,92]. We will primarily focus on the cellular and molecular functions of high-risk genes in (1) neurogenesis, including both progenitor proliferation and differentiation into neurons; (2) migration of the newborn projection neurons to the cortical plate; (3) cell specification of projection neurons, defining cortical lamination; (4) morphogenesis and synaptogenesis of projection neurons; (5) migration and development of cortical GABAergic interneurons (Figure 1, Table 1).

### 4.1. Neurogenesis

Alterations in neurogenesis can lead to both microcephaly and megalencephaly, which are frequently observed in ASD [93]. Proper neurogenesis requires different signaling pathways, including the Wnt, PI3K/AKT/mTOR, and Notch pathways. These pathways can lead to changes in gene expression programs, including transcription (e.g., *CTNBB1*, *CHD8*, *FOXP1*, *POGZ*) and mRNA translation (e.g., *CELF4*, *DDX3X*) (Figure 1A). While we will discuss these pathways and genes in relationship to cortical neurogenesis, they are also important in earlier neural patterning and later stages of brain development.

Beyond a well-established role in early brain patterning [94], the Wnt pathway modulates progenitor proliferation by inhibiting cell cycle exit and differentiation through the downstream component β-catenin [95]. Mutations in *CTNNB1*, the gene encoding for β-catenin, are associated with ASD [8], ID [96,97], and other syndromic manifestations [98] (a neurodevelopmental disorder with spastic diplegia and visual defects, OMIM 615075). Mutations or altered expression of *Ctnnb1*, even via manipulation of upstream regulators, affect cortical neurogenesis [95,99,100,101,102,103,104,105]. For example, transgenic mice expressing a constitutively active β-catenin have excessive RGCs, to the expenses of IPs population [99], and consequent enlargement of the ventricles [95]. Further, in utero silencing of the histone cell cycle regulator HIRA, an activator of β-catenin expression, induces terminal mitosis, thus reducing NPC proliferation in VZ/SVZ and IZ [100]. On the other hand, increased β-catenin levels also disrupt RGC proliferation, as observed in mice with a deletion in *Apc*, a gene necessary for β-catenin degradation [101]. In both studies, restoring β-catenin levels rescued the proliferation defects [100,101].

*CHD8* [26,27,28,37] is also part of the Wnt signaling pathway [74]. Both human and mouse *CHD8* are highly expressed in the cortex at the onset of neurogenesis (with a peak at E12 in mice) [74], and decrease with the progression of corticogenesis and in adulthood [74,106]. In line with these expression data, CHD8 is indispensable for cortical neurogenesis [74]. For example, in utero knockdown of *Chd8* with short hairpin RNAs (shRNAs) at E13 results in a decrease in progenitors from VZ/SVZ and an increase in neurons at E16, indicating a premature depletion of the NPC pool [74]. Co-expression of an shRNA-resistant *Chd8* rescued this phenotype [74]. However, several *Chd8* mutant lines have consistently shown megalencephaly [75,107,108,109], which is in line with the macrocephaly observed in CHD8 patients ([110] but see also [111]). While the megalencephalic phenotype seems contradictory with the reduced progenitor proliferation, Suetterlin and colleagues have shown that the brain overgrowth starts postnatally [75]. One of the proposed mechanisms for the role of CHD8 in cortical development is the regulation of downstream genes: CHD8 is in fact an ATP-dependent chromatin remodeler enriched in the promoters of transcriptionally active genes [71,72,73]. RNA sequencing analyses on *Chd8* mutant mice [74,109] and CHD8-depleted human progenitors [71,72] have shown that many downregulated genes are implicated in Wnt signaling [72,74,109] and/or cell cycle progression [71,74,109]. Further, *Chd8* knockdown reduces Wnt-mediated transcriptional activity in mouse NPC in a luciferase assay, and co-expression of a resistant β-catenin construct normalized Wnt activity, the alterations in the number of NPC, and the gene expression dysregulation [74]. Further, as mentioned above, genes regulated by *CHD8* are enriched in ASD risk genes [71,72,73,74,75].

*DDX3X* (DEAD-box helicase 3 X-linked) is another ASD risk gene [7,38,39,112] that has recently emerged as critical for cortical neurogenesis [113], possibly through interactions with the Wnt pathway. Mutations in *DDX3X* are associated with DDX3X syndrome (OMIM 300958), an X-linked condition that manifests with ID, brain anomalies, movement disorders, and behavioral problems, including ASD [113,114]. *Ddx3x* is highly expressed early during neurogenesis (E12.5–E14.5) in RGCs, and by E16.5 in both RGCs and neurons in the cortical plate (CP) [113]. In postnatal brains, *Ddx3x* is expressed in all cortical layers and in neurons, astrocytes, oligodendrocytes, microglia, and ependymal cells [113]. In vivo silencing of *Ddx3x* at E14.5 led to an increase of Pax6^+^ RGCs and Tbr2^+^ IPs, and a decrease in differentiated neurons. This imbalance is not caused by increased RGC apoptosis or reduced migration of the neurons to the CP, suggesting that *Ddx3x* is necessary for the differentiation of progenitors into neurons. While *Ddx3x*-depleted cortices have alterations in Wnt targets [113], a causal relationship has yet to be established. A more compelling mechanism relates to the role of DDX3X in modulating mRNA translation. First, DDX3X acts on specific mRNAs that are needed for neurogenesis, including *RAC1* (Rac Family Small GTPase 1) [113]. Second, DDX3X regulates the formation of RNA-protein granules that are thought to affect mRNA translation, as shown by the formation of ectopic granules in the mouse neural progenitor line N2A or primary neurons over-expressing DDX3X clinical mutants [113].

Another signaling pathway crucial for cell growth, proliferation, and survival is the PI3K/AKT/mTOR pathway. *PTEN* (phosphatase and tensin homolog) is a tumor suppressor gene part of this cascade [115,116]. Mutations in *PTEN* are found in individuals with ASD [8] and are associated with an autosomal dominant macrocephaly/autism syndrome (OMIM 605309). Mouse models with conditional homozygous ablation of *Pten* (*Pten*^−/−^) have provided compelling evidence for a critical role of PTEN in progenitor proliferation and brain overgrowth [117]. Recent studies in *Pten* haploinsufficient mice (*Pten*^+/−^) are calling the attention on carefully considering gene dosage when modeling ASD mutations in rodents. *Pten*^+/−^ mice display brain overgrowth that appears perinatally and is maintained throughout adulthood. This phenotype is accompanied, in early phases, by increased progenitor proliferation and excess of neurons at birth, followed by postnatal apoptosis of excess neurons and excess glia in adulthood [105]. Interestingly, there seems to be a gene dosage effect, with brain overgrowth primarily led by hyperplasia (increased cell number) in *Pten*^+/−^ mice [105] and by both hyperplasia and hypertrophia (increased cell size) in *Pten*^−/−^ mice [105,118,119]. These distinct phenotypes seem to track to distinct pathways: increased β-catenin signaling in *Pten*^+/−^ mice, again supporting the role of β-catenin in proliferation, as opposed to both increase β-catenin and mTOR activity in *Pten*^−/−^ mice [105]. Compatible with this hypothesis, brain overgrowth can be suppressed by *Ctnnb1* haploinsufficiency, but not Mtor in *Pten*^+/−^ mice [105], or by mTORC1 knockout [120] or inhibition [121] in *Pten*^−/−^ mice.

Brain organoids derived from human and mouse embryonic stem cells engineered with homozygous deletions in PTEN also support a key role for this gene in progenitor proliferation. In the human organoids, *PTEN* loss resulted in increased protein kinase B (AKT) signaling, which delayed cell cycle exit resulting in prolonged proliferation of neural progenitor, including the oRG population [122]. This led to increased organoid size, outgrowth of neuroepithelial tissue, and folding [122]. Rescue of PTEN levels or AKT inhibition in mutant organoids normalized proliferation rate and neuronal differentiation. In addition to direct mutations in *PTEN*, also manipulating upstream regulators converge on defects in proliferation. For example, Lei and colleagues [123] described the increased proliferation of Pax6^+^ RGCs in VZ and reduced NPCs exiting the cell cycle in a conditional knockout mouse for *Kdm6a* (Lysine Demethylase 6A, also known as *Utx*), a lysine demethylase implicated in ID (Kabuki syndrome, OMIM 300867) affecting *Pten* transcription. These mutant mice have increased methylation of the *Pten* promoter and, consequently, decreased PTEN expression. Re-expression of PTEN rescued the abnormalities in NPC proliferation, reinforcing the importance of this phosphatase in the regulation of NPCs proliferation and differentiation [123].

Finally, ASD risk genes are also associated with the Notch signaling pathway, which modulates fate decisions in embryonic and adult NSCs [124,125]. *FOXP1* (forkhead box P1) is part of a subfamily of transcription factors that temporally and spatially control the expression of a wide range of genes by binding to their promoter and enhancer sequences via a forkhead DNA binding motif [126]. *FOXP1* is a high-risk gene for ASD [8] and mutations in the gene result in a neurodevelopmental condition called FOXP1 syndrome (OMIM 613670; [127] and references therein). *Foxp1* expression peaks in the VZ of the mouse cortex between E10 and E12.5, and then decreases between E13.5 and E16.5 [128]. *Foxp1* knockdown by in utero electroporation (IUE) results in reduced post-mitotic cortical neurons and increased IP in the intermediate zone (IZ) [129]. In line with these findings, experiments in murine cultured NSCs found that *FOXP1* affects NSC differentiation into neurons and astrocytes by repressing Jagged1—a Notch ligand expressed in NSCs that is required for their maintenance in SVZ [129]. In vitro treatment with a Jagged1 inhibitor restores differentiation of Foxp1-depleted NSCs [129]. More recent findings indicate that *Foxp1* is needed early during neurogenesis for the maintenance of the apical RG (aRG) pool [128]. Mice overexpressing *Foxp1* had increased aRG self-renewal and their differentiation into early-born neurons at the detriment of late-born neurons and glia, while mice knocked out for *Foxp1* disrupted early-born populations, with no effects on late-born neurons [128]. These observations indicate that sustained *Foxp1* expression can extend the temporal window of early neurogenesis [128].

Another top ASD gene, *POGZ* (Pogo transposable element-derived protein with zinc finger domain; OMIM 616364; [26,27,28,37]), also favors neuronal differentiation by inhibiting the Notch signaling. IUE-mediated knockdown of *Pogz* leads to an increased proportion of NSCs, and reduced IPs and late-born neurons [130]. A murine *Pogz* rescued this phenotype, while mutants carrying ASD pathogenic mutations did not [130]. In line with these data, NSCs derived from induced pluripotent stem cells of a patient with ASD carrying a de novo missense mutation in *POGZ* also show increased proliferation and reduced degree of neuronal differentiation [130].

### 4.2. Migration

Newly generated glutamatergic neurons migrate radially from the SVZ to the preplate, situated at the pial surface. Initially, neurons acquire a unipolar morphology by extending a basal process towards the pial surface and then they translocate their nucleus and cytoplasm towards it (somal translocation). Later, neurons adopt a multipolar morphology while in the IZ that allows them to search for cell polarity cues in the environment. Then, they acquire a bipolar morphology and use the radial fiber of RGCs to migrate towards the pial surface (locomotion). At E50–75 in humans and E14 in mice, as neurogenesis progresses and more projection neurons reach the preplate, this region splits into the marginal zone and the cortical plate (CP). Postmitotic neurons continue to occupy the CP in an “inside-out” fashion, eventually forming six cortical layers (L1–L6). Early-born neurons will constitute deep layers (first L6 and later L5); late-born neurons will populate upper layers (first L4, then L3 and L2) [84,85] (Figure 1**)**.

Defects in neuronal migration can cause cortical malformations (e.g., lissencephaly, heterotopia, polymicrogyria) that are frequent in individuals with ASD [81,113,131,132,133,134,135,136]. Not surprisingly, neuronal migration is regulated by several ASD genes [106,137,138,139] (Figure 1B). Some of these genes can impact proliferation as well, making it difficult to disentangle the impact of earlier proliferation defects on migration. For example, *CHD8* regulates both proliferation (see above, [74]) and migration [106]. Interestingly, *Chd8* downregulation during embryonic stages does not totally abolish neuronal migration, but it delays it until the early postnatal period [106]. Similarly, the ASD risk gene *TCF4* (transcription factor 4) [8], associated with Pitt–Hopkins syndrome (OMIM 610954), regulates both proliferation and migration, as shown in a *Tcf4* haploinsufficient mouse model [140]. Matsumura and colleagues [130] described that knockdown of *Pogz* delayed migration to the CP, but that defect tracked down to impaired neuronal differentiation (see above). Importantly, the study also showed that mice engineered to carry a de novo missense mutation found in a patient with ASD had mislocalization of upper-layer neurons into lower layers, a condition that was maintained until adulthood. Interestingly, this mutation did not affect GABA^+^ interneurons [130].

One of the emerging complications in ASD is that some risk genes might act with both a loss and a gain-of-function mechanism [8], as in the case of FOXP1 syndrome [127]. Additionally, there are other ASD risk genes where loss-of-function and missense mutations yield different clinical severity (e.g., *DDX3X* [113]) or where mutations in the same gene segregate into distinct biological signatures (e.g., *ADNP* [141]). In the case of *Foxp1*, loss-of-function and gain-of-function mutations appear to impact cortical migration [137,142]. Mice with forebrain-specific knockout of *Foxp1* present reduced migration of late-born neurons from the SVZ/VZ to CP. The defect could be rescued by wild-type FOXP1, but not a mutant preventing sumoylation [137]. Interestingly, sumoylation modulates the interaction of FOXP1 with the chromatin remodeling complex NuRD, thus likely affecting gene expression [137]. Further, cortical migration was impacted by the expression of a *Foxp1* mutant mouse homolog to the human recurrent nonsense mutation p.Arg525* ([127] and references therein) [142]. In both human cell lines [143,144] and mouse progenitors in vivo [142], the over-expressed mutant *Foxp1* fails to reach the nucleus and form cytoplasmic aggregates. In utero expression of the mutant *Foxp1* resulted in a higher proportion of cells with multipolar morphology in the IZ, and a consequent decrease of postmitotic neurons reaching the CP [142].

Other ASD risk genes, including the BAF chromatin remodeling complex subunit *BCL11A* (MIM 617101, [6,145]) and *FMR1* (MIM 300624), are necessary for the switch of newborn neurons from multipolar to bipolar in the IZ. Failing in this process also underlies the delayed migration of late-born neurons to the upper layers in *Bcl11a*-deficient mice [139] and *Fmr1* knockout mice [138]. Impaired multipolar-to-bipolar transition is driven by increased *Sema3c* expression resulting from *Bcl11a* loss [139] or decreased *Chd2* levels in *Fmr1* knockout mice [138]. In both of these models, the genetic manipulations did not affect progenitor proliferation or differentiation, indicating that the migration phenotype was not a secondary effect of earlier alterations.

### 4.3. Laminar Specification

Laminar specification is an essential process for shaping the cortex into six layers, completed at around 7 months of gestation in humans and at postnatal day 7 in mice. With cell fate specification, the developing glutamatergic projection neurons acquire subtype specificity. Two main subtypes of excitatory neurons are generated: corticofugal and intra-telencephalic projections neurons. Corticofugal projection neurons extend their axons away from the cortex into the thalamus (corticothalamic projection neurons, mostly in L6) or subcerebral nuclei (subcerebral projection neurons, mostly in L5). On the contrary, intra-telencephalic projection neurons (mostly located in upper L2 and L3) extend their axons within the cortex, to the ipsilateral (associative projection neurons) or contralateral (commissural projection neurons) hemisphere. Most of the latter projections cross through the corpus callosum (callosal projection neurons) (Figure 1).

Cell type specification is orchestrated by a set of transcription factors [146,147,148,149,150,151,152,153,154,155,156,157,158,159] (Figure 1C). Some of these transcription factors are associated with ASD, including *BCL11A* and *TBR1* (OMIM 606053, [8,160,161]). In addition to regulating migration [139], *Bcl11a* contributes to specifying subcerebral projection neurons by repressing *Tbr1* [159]. In fact, the IUE-mediated knockdown of *Bcl11a* at E13.5 results in an increase of TBR1^+^ neurons and a reduction of BCL11B^+^ neurons in the postnatal cortex [159]. Further, *Bcl11a*-deficient mice show a reduced pyramidal tract due to defective development of the corticospinal projections, and this defect can be rescued by increasing *Bcl11b* expression or silencing *Tbr1* [159]. *Tbr1* is a major driver of corticothalamic specification [70,158,162,163,164]. In fact, L6 neurons in the cortex of *Tbr1* null mice misroute their axons to aberrantly innervate the corticospinal tract [162]. Further, L6 neurons in a mouse with conditional *Tbr1* ablation show a shift towards L5 identity, with repercussions on, among others, dendritic patterning (see below) [70]. Recently, post-transcriptional mechanisms influencing specification have also begun to emerge [78,165,166], casting a scenario where proper corticogenesis requires a fine-tuning of gene expression.

### 4.4. Morphogenesis and Synaptogenesis

During early mid-fetal development, after neurogenesis has peaked and migration has begun, dendritogenesis [167,168], axonogenesis [169], and synaptogenesis [170] ensue. Although these three processes start prenatally, they remain active until adolescence and early adulthood to refine brain circuits. In the neocortex, dendritogenesis and axonogenesis begin when a neuron is in the IZ, after leaving the VZ, and obtains multipolar morphology. At this stage, neurite polarity is determined, where one process extends tangentially within the IZ to become the axon and another process extends towards the pial surface, serving as the leading process for radial migration, and will become the apical dendrite once the neuron has migrated to the CP. Basal dendrites sprout from the soma perpendicular to the pial surface and grow towards the VZ. As the apical and basal dendrites elongate, they will arborize creating many branched processes and synaptogenesis will then begin to form (Figure 1). Throughout childhood and adolescence, long thin and filopodia-like protrusions (immature spines) will be pruned and stabilized to stubby and mushroom-like protrusions (mature spines) to refine brain circuits [170,171].

There is a large body of evidence implicating altered neuronal morphogenesis and synaptogenesis in ASD risk [171,172] (Figure 1D). ASD risk genes that have been recently implicated in axonogenesis include ankyrin 2 (*ANK2*), *CHD8*, and the dual-specificity tyrosine phosphorylation regulated kinase 1A (*DYRK1A*; OMIM 614104) [6,8,26,27,28,37]. Cultured hippocampal neurons from a mouse expressing an *Ank2* carrying an ASD-pathogenic mutation show increased axon branching, without a concomitant change in axon length or dendritic arborization [173]. The proposed mechanism suggests that *Ank2* functions in controlling axon branching by suppressing microtubule invasion into newly formed filopodia [173]. However, this did not translate into gross differences of the major axon tract, including the corpus callosum [173], but resulted in a more subtle increase in cortical connectivity. Suppression of callosal projections, on the contrary, is observed in cortices electroporated with an anti-*Chd8* shRNA [106]. One potential mechanism is the *Chd8*-dependent regulation of genes implicated in axonal development, as detected in human neural progenitor cells [72]. Furthermore, knocking down *Dyrk1a* or overexpressing it as wild-type or missense mutants homologous to those found in ASD individuals reduce the axonal length in mouse cortical neurons, revealing that the dosage of *Dyrk1a* is critical for proper neurite and axon development [174].

Dendritogenesis requires an intact expression of ASD risk genes. In addition to defective axonogenesis, *Chd8* knockdown in L2/3 pyramidal neurons results in reduced dendritic density and complexity [74,106], which could be alleviated upon induction of the Wnt signaling [74]. *ARID1B* (AT-rich interaction domain 1B; OMIM 135900, [6,8]) is necessary for the proper growth of apical and basal dendrites, as *Arid1b*-deficient cortical mouse neurons display shorter dendrites and defective orientation of basal dendrites, but not apical dendrites, [175]. *Arid1b*-deficient L2–4 pyramidal neurons show smooth apical dendrites without branches and a reduced number of primary, secondary, and tertiary apical dendrites [175]. However, these results were obtained with shRNA-mediated knockdown and were not replicated in an *Arid1b* haploinsufficient mouse [176], emphasizing again the importance of taking dosage into account. One example where dendritogenesis defects are secondary to altered cell identity relates to *TBR1*, which, as discussed above, drives corticothalamic specification in L6 [70,158,162,163,164]. L6 pyramidal neurons with conditional haploinsufficiency of *Tbr1* extend their apical dendrites to L1 and L2/3 instead of growing into L4. These defects in dendritic patterning indicate that L6 neurons have L5-like properties [70], further corroborating the importance of *Tbr1* for identity specification (see above). Dendritogenesis of L5 neurons requires, among other factors, *Foxp1*, as the embryonic expression of an ASD-relevant *Foxp1* mutant causes shorter apical dendrite in mature L5 pyramidal neurons [142]. FOXP1 sumoylation is critical for its role in dendritic morphogenesis [137,177]. For example, the ablation of *Foxp1* in rat cortical neurons results in dendritic malformation and shorter dendrites, with no change in axonal length [177]. This phenotype can be rescued with wild-type FOXP1, but not with a non-sumoylatable FOXP1 mutant [177].

As mentioned, the synaptic function is a nexus of liability for ASD [6,8,170,171]. Mutations in many risk genes encoding synaptic proteins, including scaffolding protein SynGAP (encoded by *SYNGAP1*) [178,179,180] and *SHANK3* [181,182], glutamate ionotropic receptor GluN2B (encoded by *GRIN2B*) [183], voltage-gated sodium channel Na_v_1.2 (encoded by *SCN2A*) [184], synaptic translational regulator FMRP (encoded by *FMR1*) [185,186] all result in defects in the formation and/or the maturation of the synapses. Mutations in ASD risk genes encoding transcription factors can also impact synaptogenesis. For example, heterozygous mice for an ASD-associated mutation in *Pogz* show an increased density of dendritic spines in L2/3 pyramidal neurons [130]. Mice haploinsufficient for *Tbr1* in L5 or L6 have, on the contrary, reduced density of mature excitatory and inhibitory synapses accompanied by an exuberance of immature filopodia, [70,76], indicating suppressed maturation.

### 4.5. Migration and Development of Cortical Interneurons

Cortical inhibitory interneurons develop predominantly from areas in the subpallium: the medial and caudal ganglionic eminences (MGE and CGE, respectively) of the ventral telencephalon and the preoptic region [187]. After differentiation—a process based on both place of origin and time—the interneurons migrate tangentially to target locations in the neocortex, populating all cortical layers, where they act as regulators of glutamatergic projection neurons by providing local inhibition. The most common types of interneurons in the cortex are parvalbumin (PV), somatostatin (SST), and ionotropic serotonin receptor 5HT3a (5HT3aR) cells (reviewed in [187]). Each of these subtypes has different laminar distributions, in addition to their own subgroups of cells based on morphology and function, creating a diverse array of critical cortical cell populations.

The mouse homolog of the risk gene *CHD2* (chromodomain helicase DNA binding protein 2; OMIM 615369, [8,188]) controls the genesis of cortical interneurons [189]. In the somatosensory cortex of *Chd2* haploinsufficient mice, there is a significant reduction in the density of PV and SST neurons, due, at least partially, to a reduction in interneuron progenitors expressing *Nkx2*-1 in the MGE [189]. *Nkx2*-1 is fundamental for MGE neurogenesis [190,191] and evidence in human embryonic stem cells indicates that this gene is directly regulated by CHD2 [192]. The lamination of the interneurons is altered in *Tbr1* mutants [70,77]. A mouse heterozygous for an ASD-associated missense mutation displays a disproportional distribution of PV interneurons in the deeper cortical layers [77], with no changes in other interneurons populations. However, *Tbr1* haploinsufficiency in L6 results in a similarly abnormal patterning, but only at the expense of SST interneurons [70]. The mechanisms behind these changes in the tangential migration of interneurons from deep to superficial cortical layers—and thus the reasons behind the incongruences in cell type between the two studies [70,77]—are still unknown, but might be potentially due to alterations of signaling from the excitatory signal. An astonishing example of how interneurons can be regulated by an ASD gene in a non-cell-autonomous fashion relates to *PTEN*. Wong and colleagues found that interneurons in the primary somatosensory cortex undergo cell death between postnatal days 5 and 10 [193]. Interneurons that received robust excitatory inputs from pyramidal cells at the beginning of this interval escaped cell death, indicating that pyramidal cell activity orchestrates interneuron survival [193]. This regulation requires *Pten*: (1) PTEN levels were significantly decreased in interneurons following the activation of pyramidal cells at postnatal day 8; and, (2) there was a higher density of PV and SST interneurons after postnatal day 10 in a mouse with ablation of *Pten* in postmitotic MGE interneurons or with pharmacological inhibition of PTEN [193].

While all of these ASD risk genes affect both pyramidal and interneurons populations in the developing cortex, there is one gene, *ARID1B*, which seems to affect most predominantly the birth and migration of interneurons. Haploinsufficiency of *Arid1b* in mice has been shown to suppress the generation of inhibitory interneurons both in vivo and in vitro, due to the combination of decreased proliferation and increased apoptosis of ventral progenitor cells in the MGE [176]. One proposed mechanism relates to the direct regulation of the *Pvalb* gene (encoding parvalbumin) [176]. These alterations are not accompanied by changes in the density or lamination of cortical excitatory neurons (see above for a discussion about phenotypes in *Arid1b*-deficient pyramidal neurons; [175]). In support that *Arid1b* pathophysiology depends on the interneuron populations is a recent study using mice with *Arid1b* haploinsufficiency selectively in PV or SST interneurons [194]. While both models displayed behavioral phenotypes relevant to ASD, they did have distinct traits, with the PV-specific haploinsufficient mice showing sociability and social recognition deficits, anxiety, and a depression-like phenotype, and the SST-specific haploinsufficient mice exhibiting excessive repetitive behaviors [194].

## 5. Conclusions

In this review, we argue that findings in rodent models, along with human genetic, imaging, and neuroanatomical evidence, pinpoint neocortical development as a critical spatiotemporal window for ASD liability. Most ASD risk genes are involved in multiple steps of corticogenesis, emphasizing again the heterogeneity of the risk architecture (Table 1).

Many risk genes are functionally interrelated: perturbations in one risk gene can have repercussions on the expression of other downstream risk genes. Tackling the function of such master regulators of other risk genes is a promising future avenue to understand convergences and divergences between risk genes. Further, observations in human stem cell-derived models (e.g., organoids) will be key to identify species-specific alterations that define the unique features of social behavior in humans.

## Figures and Tables

**Table 1:** Summary of the findings discussed in the review. Acronyms in the glossary.

**Gene**	**Phase**	**Model or Experimental Manipulation**	
Cortical glutamatergic neurons			
*ANK2*	morphogenesis; synaptogenesis	KI mouse	[173]
*ARID1B*	morphogenesis; synaptogenesis	shRNA-mediated KD by IUE	[175]
		shRNA-mediated KD in mouse primary cortical neurons	[175]
*BCL11A*	migration	KO mice	[139]
	specification	shRNA-mediated KD by IUE	[159]
*CHD8*	neurogenesis	shRNA-mediated KD by IUE	[74]
		KO mice	[75,107,108,109]
		shRNA-mediated KD in hNPCs	[71,72]
	migration	shRNA-mediated KD by IUE	[65]
	morphogenesis; synaptogenesis	shRNA-mediated KD by IUE	[74,106]
		shRNA-mediated KD in hNPCs	[72]
		shRNA-mediated KD in mouse primary cortical neurons	[106]
*CTNNB1*	neurogenesis	KI mouse	[95]
		KO mice	[99,101,103]
		cDNA-mediated KI by IUE	[99]
		shRNA-mediated KD IUE	[100,101]
		primary cortical neurons from KO mouse	[99,103]
		shRNA-mediated KD in mouse NPCs and cell lines	[100]
		cDNA-mediated KI in mouse primary cortical neurons	[104]
*DDX3X*	neurogenesis	sgRNA- and siRNA-mediated KD by IUE	[113]
*DYRK1A*	morphogenesis; synaptogenesis	cDNA-mediated KI by IUE	[174]
*FMR1*	migration	KO mice	[138]
	morphogenesis; synaptogenesis	KO mice	[185,186]
**Gene**	**Phase**	**Model or Experimental Manipulation**	
*FOXP1*	neurogenesis	KI mice	[128]
		shRNA-mediated KD by IUE	[129]
		shRNA-mediated KD in mouse primary cortical neurons	[129]
	migration	KO mice	[137]
		cDNA-mediated KI by IUE	[142]
		cDNA-mediated KI in mouse primary cortical neurons, hNPCs and neurospheres	[137]
	morphogenesis; synaptogenesis	KO mice	[137]
		cDNA-mediated KI IUE	[142]
		cDNA-mediated KI in mouse primary cortical neurons, hNPCs and neurospheres	[137]
		shRNA-mediated KD in mouse primary cortical neurons	[177]
*POGZ*	neurogenesis	shRNA-mediated KD by IUE	[130]
		KI mice	[130]
		hNPCs	[130]
	migration	shRNA-mediated KD by IUE	[130]
		KI mice	[130]
	morphogenesis; synaptogenesis	KI mice	[130]
*PTEN*	neurogenesis	KO mice	[105,117,118,119,120,121]
		shRNA-mediated KD by IUE	[123]
		sgRNA-mediated KO in human organoids	[122]
*SCN2A*	morphogenesis; synaptogenesis	KO mice	[184]
*SHANK3*	morphogenesis; synaptogenesis	KO mice	[181]
		sgRNA-mediated KO in mice	[181]
*SYNGAP1*	morphogenesis; synaptogenesis	KO mice	[180]
*TBR1*	specification	KO mice	[70,158,163,164]
	morphogenesis; synaptogenesis	KO mice	[70,76,164]
		primary cortical neurons from KO mouse	[76]
*TCF4*	neurogenesis;	KO mice	
	migration;		[140]
	morphogenesis; synaptogenesis		
Cortical interneurons			
*ARID1B*	proliferation; lamination; synaptogenesis	KO mice	[176]
*CHD2*	proliferation	KO mice	[189]
*PTEN*	survival	KO mice	[193]
*TBR1*	lamination	KO mice	[70,77]

## Glossary

ADHD	attention deficit hyperactivity disorder
aIP	apical IP
ASD	autism spectrum disorder
bIP	basal IP
CGE	caudal ganglionic eminence
CNV	copy number variation
CP	cortical plate
GWAS	genome-wide association studies
ID	intellectual disability
IP	intermediate progenitor
IUE	in utero electroporation
IZ	intermediate zone
KD	knockdown
KI	knockin
KO	knockout
L1-6	cortical layers 1-6
MGE	medial ganglionic eminences
NPCs	neural progenitor cells
NSCs	neural stem cells
OMIM	Online Mendelian Inheritance in Man (https://www.omim.org)
oRG	outer radial glia
oSVZ	outer subventricular zone
PV	parvalbumin
RGC	radial glial cells
scRNAseq	single-cell RNA sequencing
shRNA	short hairpin RNA
SNP	single-nucleotide polymorphism
SNV	single-nucleotide variation
SP-IZ	subplate-intermediate zone
SST	somatostatin
SVZ	subventricular zone
VZ	ventricular zone
WES	whole-exome sequencing
